# Travel restrictions and variants of concern: global health laws need to reflect evidence

**DOI:** 10.2471/BLT.21.287735

**Published:** 2022-03-01

**Authors:** Benjamin Mason Meier, Judith Bueno de Mesquita, Gian Luca Burci, Danwood Chirwa, Stéphanie Dagron, Mark Eccleston-Turner, Lisa Forman, Lawrence O Gostin, Roojin Habibi, Stefania Negri, Alexandra Phelan, Sharifah Sekalala, Allyn Taylor, Pedro A Villarreal, Alicia Ely Yamin, Steven J Hoffman

**Affiliations:** a Gillings School of Global Public Health, University of North Carolina at Chapel Hill, Chapel Hill, United States of America (USA).; b School of Law and Human Rights Centre, University of Essex, Colchester, England.; c Graduate Institute of International and Development Studies, Geneva, Switzerland.; d Faculty of Law, University of Cape Town, Cape Town, South Africa.; e Faculties of Law and Medicine, Global Studies Institute, University of Geneva, Geneva, Switzerland.; f Department of Global Health & Social Medicine, King's College London, London, England.; g Dalla Lana School of Public Health, University of Toronto, Toronto, Canada.; h O’Neill Institute for National & Global Health Law, Georgetown University Law Center, Washington, DC, USA.; i Global Strategy Lab, York University, 2120 Dahdaleh Building, 4700 Keele Street, Toronto, Ontario, M3J 1P3, Canada.; j School of Law, University of Salerno, Fisciano, Italy.; k Center for Global Health Science and Security, Georgetown University, Washington, DC, USA.; l School of Law, University of Warwick, Coventry, England.; m School of Law, University of Washington, Seattle, USA.; n Max Planck Institute for Comparative Public Law and International Law, Heidelberg, Germany.; o Harvard Law School and Harvard TH Chan School of Public Health, Cambridge, USA.

As the coronavirus disease 2019 (COVID-19) spread in the early days of the pandemic, governments neglected World Health Organization (WHO) guidance and imposed travel restrictions. These public health measures employed varied levels of restrictiveness at national borders, in some cases banning all travel between countries. Where these border control measures were undertaken for domestic political reasons, enacted without consideration of public health evidence, they divided the world when solidarity was needed most.^1^ Such measures undermined global health law that countries have established as a foundation for preventing and responding to public health emergencies of international concern.

With the emergence of the Omicron variant, national governments once again returned to international travel restrictions, posing challenges for the rule of law in global health governance. Future reforms of global health law must account for this continuing impulse to enact travel restrictions, ensuring that international legal obligations reflect evolving public health evidence.

The *International Health Regulations, 2005 revision*; IHR (2005) govern how countries address collective threats in global solidarity; yet international travel bans can drive countries apart through economic isolation, trade disruptions, discriminatory restrictions and rights violations.^2^ Fearing that government actions would undermine the IHR at the start of the COVID-19 pandemic, the Global Health Law Consortium in February 2020 examined the legality of targeted travel restrictions.^3^

In the early COVID-19 response, we remained concerned where national governments bypassed WHO’s public health recommendations in a rush to impose travel bans that targeted specific countries in ways that exacerbated political divisions, blocked essential goods and deflected from established mitigation measures – including travel advisories, diagnostic testing and quarantine policies.^4^ Although such travel restrictions were thought to be epidemiologically ineffective against past infectious disease threats, select countries used these measures to slow importation of COVID-19 in specific contexts.^5^ It is now clear, in accordance with the precautionary principle, that travel restrictions can be legally justified under certain conditions if based on evolving scientific evidence and if less restrictive alternatives are not feasible.

We continue to be concerned, however, that many governments are still reflexively deploying discriminatory travel restrictions to meet domestic political imperatives, prioritizing government action without sufficient justification in public health evidence. The latest round of travel restrictions, enacted in response to the Omicron variant, reveal the often-pernicious effects of such decision-making on low- and middle-income countries. When South Africa transparently reported a new variant of concern, countries immediately limited travel to and from South Africa, in some cases expansively targeting additional Southern African countries, without consideration of WHO guidance and despite updated evidence of variant spread well beyond the targeted countries.^6^

The mixed public health success of travel restrictions during the pandemic calls into question IHR (2005) obligations in the context of evolving public health knowledge.^7^ The IHR seek to frame responses to public health emergencies while avoiding unnecessary or disproportionate interference with international traffic, recognizing that travel bans have often shown limited effectiveness, discouraged outbreak reporting and undermined humanitarian responses. Under the obligations of the IHR (2005), national health measures “shall not be more restrictive of international traffic … than reasonably available alternatives” and shall be implemented “with full respect for the dignity, human rights and fundamental freedoms of persons”. International travel restrictions are not prohibited by the IHR in all circumstances, but any restrictions must be based on scientific principles and WHO guidance.^8^ These IHR assessments require evidence to understand where travel restrictions can be necessary and proportionate.

In reforming global health law to reflect evolving public health knowledge, IHR (2005) revisions and pandemic treaty negotiations must provide flexibility in implementing evidence-based travel restrictions while strengthening WHO guidance to reflect epidemiologic data, facilitate health equity and support international cooperation.^9^^,^^10^ Achieving these goals will require greater empirical understanding of the necessity and proportionality of varied types of travel restrictions and differentiated standards across national contexts.
